# The Shoe Fits, but the Footprint is Larger than Earth

**DOI:** 10.1371/journal.pbio.1001701

**Published:** 2013-11-05

**Authors:** William E. Rees, Mathis Wackernagel

**Affiliations:** 1 University of British Columbia, School of Community and Regional Planning, Vancouver, British Columbia, Canada; 2 Global Footprint Network, Oakland, California, United States of America; University College London, United Kingdom

## Abstract

In a Formal Comment, Rees and Wackernagel defend their global Ecological Footprint calculations against criticisms in this issue of *PLOS Biology* that they are potentially misleading.

In their Perspective in this issue of *PLOS Biology*, Blomqvist et al. [1] set out to demonstrate that “Ecological Footprint measurements, as currently constructed, are so misleading as to preclude their use in any serious science or policy context.” Should the reader be confident in this assessment or are Ecological Footprint methods and results adequate to guide sustainability policy?

Their Perspective, “Does the Shoe Fit?”, does not question the fundamental purpose of Footprint accounting. The method is designed to estimate human demand for biocapacity, defined as: ‘the aggregate area of land and water ecosystems required by specified human populations to produce the ecosystems goods and services they consume and to assimilate their carbon wastes.’ Footprint accounting is thus based on the premise that the regenerative capacity of the ecosphere is associated with productive ecosystem area. The production of food and fibre; the urbanization of once agricultural or forested lands; and the sequestration of that portion of carbon emissions from fossil fuels that is not already absorbed by oceans or by long-term sequestration strategies in agriculture or forestry, all constitute competing or non-overlapping uses of ecosystems. (Typically, one cannot simultaneously use paved-over land for food production or forest products; today's cropland and commercial forests are usually carbon sources, not sinks). We estimate and sum these separate areas to estimate study populations' total Ecological Footprints.

Global Footprint Network has developed national Footprint accounts, using consistent United Nations data sets to provide both Footprint and biocapacity assessments of most nations [2]–[4]. One can therefore readily compare national and global Footprints with domestic and global supplies of biocapacity, respectively. This comparison helps to shed light on core research questions fundamental to human wellbeing and sustainability: How does a population's consumption-based demand compare to its domestic biocapacity? Is the population dependent on local overuse, net-imports, or net-appropriations from the global commons? How do various nations/populations compare and what are the trends over time? At the global scale, is *H. sapiens* living within the regenerative means of nature?

Global Footprint Network's most recent accounts reveal that Earth's biocapacity in 2008 was 12 billion hectares (ha) compared to humanity's Footprint of 18.2 billion ha, and that the average Ecological Footprint had reached 2.7 global hectares (gha) per capita compared to only 1.8 gha of available biocapacity per capita [5]. (A global hectare is a hectare of global average productivity.) This difference means that humanity is in ecological overshoot, currently using *at least* 50% more of nature's goods and services than ecosystems regenerate [5]–[7]. These national Footprint estimates are conservative since data limitations prohibit consistent adjustments to account reliably for the over-exploitation of ecosystems (see below), and carbon dioxide, mainly from fossil fuel and cement production, is the only waste stream considered. They nevertheless constitute *the most comprehensive assessments of the ecological status of nations available*.

Independents tests of the method are, of course, essential. In the past few years over a dozen national and international government agencies have reviewed the National Footprint Accounts for their countries (see www.footprintnetwork.org/reviews). The French Ministry of Sustainable Development, for example, independently recalculated the French Footprint from 1961 to today obtaining results within 1–3 percent of Global Footprint Network's assessments [8].

## The Writers' Diversion

Blomqvist et al. ignore national and regional Footprint assessments on grounds that these merely assess “self-sufficiency”. Instead they jump straight to the global scale.

This is problematic. National/regional Footprint assessments reveal some of the most significant findings of Footprint accounting and those with the highest policy utility. Even relative “self-sufficiency” is becoming an increasingly important policy consideration in an era of accelerating global change as revealed by rapid growth in international ‘land-grabbing’ by countries with ecological deficits [9]. Also, most policy-oriented Footprint studies are carried out at the city, regional, and national levels because they correspond to levels of government that have authority to act. (There is no corresponding global authority.) The method currently shows that about 84% of the world population live in countries that run growing ecological deficits, i.e., they are living on overuse of their own ecosystems, on imported biocapacity or on appropriations from the global commons [4],[9]. Only a few countries have significant biocapacity reserves and these resources are largely already taken up in satisfying the deficits of net-importing countries. For example, the domestically unused half of Canada's cropland, commercial forest land, and fisheries are presently committed to export markets [10],[11]. Whether running an ecological deficit in an uncertain world already in overshoot is a significant risk every nation must evaluate for itself. However, there is nothing gained by *not* knowing one's country's biocapacity balance, and there are presently no better estimates than those delivered by Global Footprint Network's current Footprint accounts.

## What Footprint Estimates Measure

Many of Blomqvist et al.'s claims are themselves misleading. For instance, they assert that “… the ecological footprint is practically equal to the carbon footprint”. This is incorrect. The carbon Footprint is only a small fraction of the domestic Footprints of many countries (e.g., 7% in the case of Tanzania in 2008) and constitutes just 55% of the total human Footprint in 2008 (up from 35% in 1961) [4],[5].

These authors also note that “none of the five non-carbon land-use categories has any substantial ecological deficit – suggesting that the depletion of cropland, grazing land, forest land, and fishing grounds is not occurring on an aggregate, global level.” In interpreting this observation, the reader must consider three facts. First, the global Footprint of cropland, built-up land, and grazing land *as currently measured* can only be less than or equal to the respective biocapacity. Unlike nations and regions, Earth cannot ‘import’ cropland biocapacity and therefore cannot show a deficit; given a total of 4.5 billion hectares of active cropland, one cannot harvest from 5.5 billion ha. Second, current cropland, forest land, and marine Footprints do not, in fact, reflect depletion but this does not imply that there is none. We fully recognize that local ecosystem abuse is a significant problem and that Footprint accounts should reflect biocapacity losses due to land/soil degradation and over-fishing. If we could apply this ‘fix’, it would certainly increase our estimates of ecological deficits. However, to make reliable adjustments would require globally consistent data sets, which do not exist. It is worth noting in passing that this lack suggests an important global-level policy recommendation, namely, the world's nations should commit to assessing land/ecosystem degradation using standardized methods to enable us to apply a ‘sustainability factor’ to our present eco-Footprint estimates. The third and final fact is that there is no inconsistency between the existence of reserves in some *global* biocapacity categories and the fact of *local* ecosystem degradation. Total biocapacity can exceed the harvest Footprint even though the exploitation of various fish stocks or forest ecosystems exceeds local regenerative capacity.

The situation is different for the carbon Footprint. We have relatively strong national and global data on carbon dioxide emissions, mostly from burning fossil fuel and cement production; accumulations in the atmosphere show unequivocally that emissions far exceed the sequestration capacity of the ecosphere. We also have acceptable estimates of sequestration rates by average forest ecosystems based on an extensive literature review, on Food and Agriculture Organization and Intergovernmental Panel on Climate Change reports (approximately1 metric ton carbon per ha per year [12]). We can thus readily estimate the global carbon Footprint and demonstrate overshoot.

While Blomqvist et al. do not contend the global carbon-sink deficit (the only part of the Footprint accounts they review), they seem to dismiss its relevance on grounds that if the estimated natural carbon sequestration rate were increased to 2.6 t ha^−1^ yr^−1^, *“the entire global ecological overshoot disappears”* and, following on, that technological fixes such as wind and solar energy might also reduce or offset carbon emissions. These assertions warrant two comments. First, while spatial and temporal variation in carbon sequestration is significant, our carbon Footprint is based on current best estimates of *de facto* average sequestration rates, not on what might be the case if the world's forests consisted of managed high-yield forest plantations. In any case, the carbon sequestration rate is not 2.6 t ha^−1^ yr^−1^ or no carbon dioxide would be accumulating in the atmosphere. Second, the criticisms made by Blomqvist's et al. indirectly suggest policy recommendations based on global Footprint results (despite their claim that the Footprint is not policy relevant); namely, develop dedicated carbon sink forest plantations of fast growing species (with a sequestration rate of, say, 2.6 ha^−1^ yr^−1^) and a crash program of renewable energy development to help eliminate the present carbon emissions overshoot. Of course, with the implementation of such programs, future Footprint assessments would show the corresponding deficit reduction.

## Conclusions

Despite Blomqvist et al.'s reservations, Footprint results show that: (1) most countries are in ecological deficit, increasingly dependent on potentially unreliable trade in biocapacity; (2) humanity is at or beyond global carrying capacity for key categories of consumption, particularly agriculture (factoring in soil loss and ecosystem degradation would reveal additional deficits); (3) global carbon waste sinks are overflowing; and (4) the aggregate metabolism of the human economy exceeds the regenerative capacity of the ecosphere (and the ratio is increasing). Significantly, Blomqvist et al. indirectly suggest several globally relevant policies to improve Footprint accounts and to reduce humanity's carbon footprint. How, in this light, the authors of the Perspective can conclude that the results of Footprint estimates are “…so misleading as to preclude their use in any serious science or policy context” remains unclear. What could we possibly gain by ignoring Footprint assessments in national and global policy-making?
